# P198: Antimicrobial treatment for urinary tract infection (UTI) among patients having total hip (THA) or total knee arthroplasty (TKA)

**DOI:** 10.1186/2047-2994-2-S1-P198

**Published:** 2013-06-20

**Authors:** L Herwaldt, S Bailin, B Johannsson, N Noiseux, A Haleem, S Johnson

**Affiliations:** 1 Internal Medicine, U of Iowa College of Medicine, Iowa City, IA, USA; 2 University of Iowa College of Medicine, Iowa City, IA, USA; 3 Orthopaedics, U of Iowa College of Medicine, Iowa City, IA, USA; 4 Pharmaceutical Care, U of Iowa Hospitals and Clinics (UIHC), Iowa City, IA, USA

## Introduction

Patients (pts) having THA or TKA were screened preoperatively (preop) and postoperatively (postop) for UTI with urinalysis (UA) and microscopic exam (micro), regardless of symptoms (sx).

## Objectives

To assess the association between UA and micro results and antimicrobial treatment (Rx) for UTI in pts having THA or TKA.

## Methods

We reviewed records of pts who had THA (n=100) or TKA (n=100) between 21/2/2011-29/6/2011 and we interviewed 50 pts who had THA or TKA between 21/5/2012-17/7/2012 to assess the association between sxs of UTI and antimicrobial Rx. We used logistic regression to identify variables associated with antimicrobial Rx for UTI.

## Results

190/200 (95%) pts had UAs, 91% had micros and 0.5% had urine cultures preop. 37 (18.5%) pts received antimicrobials for UTI preop. Positive leukocyte esterase (LE; p<.0001) and white blood cell (WBC) count > 5 (p=.0098) were associated with antimicrobial Rx for UTI preop. 198 (99%) pts had UAs, 98% had micros, and 2.5% had urine cultures after Foley removal. 72 (36%) pts received postop antimicrobials for UTI. Positive (+) LE (p<.0001), WBC count >5 (p=.014) and older age (p=.014) were associated with antimicrobials for UTI postop. Rx for UTI was related to LE level (p<.0001). 43/72 (59.7%) pts Rxed for UTIs postop did not meet criteria for UTI. 28/50 (56%) pts interviewed had sxs consistent with UTI but pts with sxs were not Rxed more often than pts without sx. 3/250 pts (1.2%) had *C difficile* infection (CDI). On the basis of the data, practice was changed. Urine cultures are obtained from pts w/ + LE and + nitrite or with sx of UTI. Pts with + cultures are treated for UTI.

## Conclusion

45.5% pts received antibiotics preop or postop for UTI; most did not meet criteria for UTI. LE results determined whether pts were Rx for UTI. Antimicrobial use and CDI among pts having THA or TKA could be reduced if only pts with UTI sxs are screened or if all pts are screened but only pts with + LE, nitrite, and cultures are treated.

## Disclosure of interest

None declared

